# Predicting unmet activities of daily living needs among the oldest old with disabilities in China: a machine learning approach

**DOI:** 10.3389/fpubh.2023.1257818

**Published:** 2023-09-12

**Authors:** Kun Wang, Jinxu Zhao, Jie Hu, Dan Liang, Yansong Luo

**Affiliations:** ^1^Zhongnan University of Economics and Law (School of Philosophy), Wuhan, Hubei, China; ^2^Nankai University (Zhou Enlai School of Government), Tianjin, China; ^3^Wuhan University (School of Physics and Technology), Wuhan, Hubei, China; ^4^Tongji Medical College of Huazhong University of Science and Technology (School of Medicine and Health Management), Wuhan, Hubei, China

**Keywords:** unmet needs, oldest old, ADL, machine learning, Shap

## Abstract

**Background:**

The ageing population in China has led to a significant increase in the number of older persons with disabilities. These individuals face substantial challenges in accessing adequate activities of daily living (ADL) assistance. Unmet ADL needs among this population can result in severe health consequences and strain an already burdened care system. This study aims to identify the factors influencing unmet ADL needs of the oldest old (those aged 80 and above) with disabilities using six machine learning methods.

**Methods:**

Drawing from the Chinese Longitudinal Healthy Longevity Survey (CLHLS) 2017–2018 data, we employed six machine learning methods to predict unmet ADL needs among the oldest old with disabilities. The predictive effects of various factors on unmet ADL needs were explored using Shapley Additive exPlanations (SHAP).

**Results:**

The Random Forest model showed the highest prediction accuracy among the six machine learning methods tested. SHAP analysis based on the Random Forest model revealed that factors such as household registration, disability class, economic rank, self-rated health, caregiver willingness, perceived control, economic satisfaction, pension, educational attainment, financial support given to children, living arrangement, number of children, and primary caregiver played significant roles in the unmet ADL needs of the oldest old with disabilities.

**Conclusion:**

Our study highlights the importance of socioeconomic factors (e.g., household registration and economic rank), health status (e.g., disability class and self-rated health), and caregiving relationship factors (e.g., caregiver willingness and perceived control) in reducing unmet ADL needs among the oldest old with disabilities in China. Government interventions aimed at bridging the urban–rural divide, targeting groups with deteriorating health status, and enhancing caregiver skills are essential for ensuring the well-being of this vulnerable population. These findings can inform policy decisions and interventions to better address the unmet ADL needs among the oldest old with disabilities.

## 1. Introduction

As China’s population ages, the country faces a significant challenge in meeting ADL needs of its older persons with disabilities. According to the 2021 seventh census, 264 million people, or 18.7% of China’s total population, are aged 60 or above^1^. With an aging population, there is an increase in the number of older persons with disabilities, which can be attributed to longer life expectancies and the widespread prevalence of chronic diseases (1, 2). Data from the Research Group of the China Research Center on Aging revealed that in 2010, there were 22.15 million older persons with partial disabilities and 10.84 million older persons with significant disabilities in China, accounting for 12.75 and 6.25% of the total older population, respectively (3). It is estimated that by 2050, China will have 27 million disabled individuals aged 65 or older (4). Consequently, an increasing demand for long-term care, which serves as an essential safeguard for the quality of life and functioning of the older persons with disabilities (5), has emerged to address the surge in assistance with ADL needs. Unmet ADL needs occur when assistance is not provided or is inadequate (6).

In comparison to the considerable demand for care, the supply of long-term care in China is significantly insufficient, leading to a large number of older persons with unmet ADL needs. Institutional stagnation and the influence of Confucian culture have resulted in informal care provided by families remaining the primary source for addressing the care needs of older persons with disabilities (7, 8). However, changes in family structure and the professionalization of women have gradually diminished families’ caregiving capacities (9, 10). Concurrently, formal care services, such as community care and institutional care, have not developed sufficiently to provide specialized care (8). This failure to obtain adequate assistance leads to a situation of unmet need (11). A recent study indicated that more than 50% of Chinese older persons with disabilities experience unmet ADL needs (12). Unmet ADL needs not only jeopardize older persons’ health but also further increase the burden on social healthcare systems. To some extent, long-term care for older persons with disabilities has become a pressing issue in Chinese society (13).

Identifying the factors affecting unmet ADL needs is crucial for improving the care system and maintaining the health of older individuals with disabilities. On one hand, unmet needs are a key concern in the development of new care systems (14). They serve as both an essential indicator for evaluating the effectiveness of existing long-term care policies and a valuable source of information for analyzing the size and characteristics of groups not covered by the current care system. Gaining insight into the unmet needs of older persons with disabilities will provide valuable guidance for future care (15). On the other hand, unmet needs can pose serious health risks to older persons with disabilities, resulting in a greater likelihood of hospital admissions, readmissions, emergency admissions, and depression (16–19). Providing adequate care can help prevent further deterioration in the health of older people with disabilities.

Previous studies have analyzed the factors affecting unmet ADL needs, demonstrating that low educational status, low income, absence of a spouse, living alone, and ADL disabilities are associated with unmet ADL needs (20–22). Some researchers have begun to focus on the caregiving situation of older persons with disabilities in China. They have utilized large-scale data from the CLHLS, which covers various aspects of the older population, such as their health status, living conditions, and economic situation. These studies have also carefully considered feature selection to identify characteristics closely associated with the risk of unmet care needs. For instance, Zeng et al. conducted an analysis based on the CLHLS database, examining the changes in health status among Chinese oldest old between 1998 and 2008. They found that advances in medication, lifestyle, and socioeconomics might compress ADL disabilities (1). Another study by Zhu analyzed factors influencing unmet long-term care needs among the Chinese oldest old. It revealed that the risk of unmet long-term needs largely depends on the economic status of the oldest old and the willingness of caregivers to provide care (13). These studies shed light on the caregiving challenges faced by the oldest old in China and highlight the importance of addressing economic factors and caregiving willingness to ensure adequate care for this vulnerable population.

While previous studies offer important findings, there are still some gaps that require additional investigation. Firstly, there is a lack of attention to the Chinese context. With a few exceptions mentioned above, the majority of studies are based on Western settings (12). China and Western countries have significant differences in their care systems for older persons. Compared to the more developed formal care service systems in Western countries, China’s formal care services are still in their early stages, with the primary responsibility for caring for older adults falling on families. There is a lack of specialized and professional care available in China (7, 8). Furthermore, many Western countries have relatively sound medical insurance systems that offer a certain level of financial support and security for older persons with disabilities. However, China’s medical insurance system provides limited financial support for older persons with disabilities (23). Additionally, in Western culture, individual respect and autonomy are highly valued, and older persons with disabilities are typically encouraged to retain their autonomy whenever possible. They are encouraged to participate in care decisions and choose care options that align with their personal needs and preferences. However, in China, the focus on this aspect is relatively weak. Secondly, there is a scarcity of analysis regarding the unmet ADL needs of the oldest old with disabilities (13). Oldest old generally refers to the oldest age group among the older population, but it does not have a globally standardized age criterion, and the specific age thresholds may vary among different studies, organizations, or countries. For instance, some studies consider individuals aged 80 and above as the oldest old (1, 13), while others define the oldest old as those aged 85 and above (24). Based on existing research on the oldest old population in China (1, 13), we have defined the oldest old as individuals aged 80 and above^2^.While some older persons may enjoy excellent health, many others still experience health challenges and an increased need for long-term care (25). Compared to other groups, older people with disabilities are more likely to experience unmet needs (26). In the context of limited care resources, addressing the unmet ADL needs of the oldest old with disabilities should be a priority. Thirdly, there are methodological limitations in using traditional linear models. The performance of traditional linear regression methods is often constrained due to their inability to handle high-dimensional and non-linear data. Moreover, traditional methods rely on strong assumptions, which are often inaccurate in real-world data (27).

As an important branch of artificial intelligence, machine learning offers superior predictive power compared to traditional linear models (28). Machine learning offers several advantages over traditional linear models: it is better at handling high-dimensional data; more flexible and versatile in terms of prediction functions; capable of handling redundant variation between highly correlated variables; and it transcends previous constraints related to prior distribution assumptions (29, 30). Due to its strong predictive performance, machine learning is widely employed in various fields (31), enabling the development of predictive tools related to healthcare (32). In fact, scholars have utilized machine learning to demonstrate promising predictive effects on different diseases (33, 34). Furthermore, the combination of machine learning and SHAP facilitates the analysis of the ranking of each predictor variable’s importance regarding its impact on the outcome.

Unmet ADL needs for the oldest old with disabilities essentially constitute a prediction problem, and the application of machine learning is both necessary and feasible. Identifying the factors that affect unmet ADL needs for the oldest old with disabilities involves determining which variables can accurately predict unmet needs. In this regard, machine learning methods with superior predictive power are more suitable than traditional linear models for analyzing the factors affecting unmet ADL needs among the oldest old with disabilities.

However, to the best of our knowledge, there are no studies that have employed machine learning to analyze the influencing factors affecting unmet ADL needs among the oldest old with disabilities in China. In this study, we utilized six machine learning methods—Naïve Bayes, Logistic Regression, Decision Tree, K-Nearest Neighbors (KNN), Random Forest, and Gradient Boosting—to identify the factors influencing unmet ADL needs among the oldest old with disabilities in China. Naïve Bayes is a probabilistic statistical classifier based on Bayes’ theorem, which assumes that features are independent of each other. Logistic Regression is a widely used linear model for binary classification problems, which predicts outcomes by fitting the relationship between features and probabilities. Decision Tree is a feature-based tree-like structure that classifies or predicts instances through a series of decision conditions. KNN model determines the category of a new sample by measuring the distance between different samples. Random Forest is an ensemble learning method that combines the predictions of multiple decision trees to improve model accuracy. Gradient Boosting is an iterative ensemble learning method that builds a powerful predictive model by progressively optimizing the prediction results. By comparing the performance of these methods, we aim to determine the most effective approach for analyzing the influencing factors affecting unmet ADL needs in this population. Our findings will contribute to a better understanding of the key factors that impact unmet ADL needs, thereby informing the government’s formulation of appropriate public policies and the enhancement of caregiver knowledge and skills.

## 2. Methods

### 2.1. Study design

The data for this study were obtained from the Chinese Longitudinal Healthy Longevity Survey (CLHLS). The survey was organized and executed by the Centre for Healthy Ageing and Development Research at Peking University and the National Development Research Institute. The CLHLS started with a baseline survey in 1998 and has conducted follow-up surveys in 2000, 2002, 2005, 2008, 2012, 2014, and 2017–2018. All participants signed a written informed document and agreed to participate in the survey project. The CLHLS is well-representative, covering 23 provinces in China, with 15,874 older persons aged 65 and over interviewed in the most recent follow-up survey (2017–2018).

**Figure 1:**
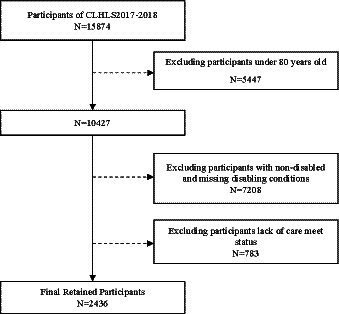
Participant Selection Flowchart.

Using the 2017–2018 CLHLS data, a total of 2,436 oldest old aged 80 and above with disability were selected for this study. Following studies using the CLHLS, we considered older persons with disabilities as individuals who required assistance in their daily living activities for more than 3 months (35). CLHLS asked participants if they needed assistance with six activities: bathing, dressing, going to the toilet, indoor activities, continence, and eating. Participants who answered yes were then asked how long they had needed assistance. Those individuals who required assistance with any of these activities for up to 3 months were considered to be older persons with disabilities and were included in the study sample. The specific data screening process is shown in Figure 1.

### 2.2. Outcome variable

Unmet ADL needs were measured by participants’ self-response to the question, “Do you think you currently get enough help with these six daily activities of bathing, dressing, going to the toilet, indoor activities, continence, and eating to meet your needs?” Participants’ responses were categorized as “completely satisfied,” “average,” and “not satisfied.” In line with existing studies (13, 36), we combined the “average” and “unsatisfied” categories. This was done for two reasons: firstly, the study aims to explore the factors influencing unmet needs among the oldest old with disabilities, and both “average” and “unsatisfied” responses indicate a level of unmet needs that may require external assistance to improve their care; secondly, only 20 older persons in the sample reported being “not satisfied,” accounting for less than 1% of the total participants, and the combined effect had minimal impact on the results. Consequently, the outcome variables were divided into two categories: fully met (coded as 1) and unmet (coded as 0). Although this measurement may contain some subjective bias, there is substantial evidence supporting its validity as an indicator of unmet ADL needs (37).

### 2.3. Predictor variables

Multiple dimensions influence unmet ADL needs in older persons. Drawing from existing studies and considering data availability (13, 36), this study selected 17 predictor variables across five dimensions: demographic, socioeconomic, health status, family relationship, and caregiving relationship. To address the small number of missing values in the predictor variables, we employed the missForest method. The missForest method has several advantages. Firstly, it can handle missing values in both numeric and categorical data simultaneously. Compared to other traditional imputation methods, missForest generally performs better in handling missing data. Additionally, missForest takes into account the correlations between variables, which allows it to perform well even in situations with a high amount of missing data or lower data quality (38).

1. Demographic characteristic variables included gender, age, and marital status. Gender is categorized as male or female. Age represents the participant’s actual age. Marital status is divided into two categories: married (including married or partnered) and unmarried (encompassing never married, divorced, separated, or widowed).
2. Regarding socioeconomic characteristics, we examined household registration, educational attainment, pension, economic satisfaction, and economic rank variables. Household registration is categorized as urban or rural. Educational attainment is divided into primary school and below or junior high school and above. Pension is classified as either yes or no. Economic satisfaction is assessed based on whether the respondent feels their financial resources are sufficient, with yes or no responses. Economic rank represents the respondent’s economic status within their local area, spanning five levels from very poor to very rich.
3. Health status variables encompassed self-rated health and disability class. Self-rated health refers to the participant’s self-assessment of their health status on a 5-point scale, ranging from very unhealthy to very healthy. Disability class indicates the severity of the participant’s disability: assistance required for 1–2 ADL tasks indicates low-level disability; 3–4 ADL tasks, medium-level disability; and 5–6 ADL tasks, high-level disability.
4. For the family relationship characteristic variables, we considered the number of children, financial support given to children, financial support received from children, and living arrangement. The number of children refers to the total number of children the participant has. Giving financial support to children denotes whether the participant provided financial support to their children in the past year, with yes or no responses. Receiving financial support from children indicates whether the participant received financial support from their children in the past year, also with yes or no responses. Living arrangement is represented by a dummy variable, divided into two categories: living with children and not living with children.
5. Regarding caregiving relationship characteristics, we selected three variables: perceived control, primary caregiver, and caregiver willingness. Perceived control refers whether the participants believe they have influence over their personal matters, with yes or no responses. Primary caregivers are categorized into three groups: spouse, children, and other. Caregiver willingness pertains to the primary caregiver’s willingness to provide care, with responses being yes or no.

### 2.4. Machine learning classifiers

In this study, we applied six machine learning methods to predict unmet ADL needs among the oldest old with disabilities: Naïve Bayes, Logistic Regression, Decision Tree, KNN, Random Forest, and Gradient Boosting.

1. Naïve Bayes is a widely used classification model based on Bayesian probability theory. It calculates the conditional probabilities of different independent features to classify categories. Though effective and capable of handling multi-category problems with small data amounts, Naïve Bayes is sensitive to data input methods.
2. Logistic Regression, a special case of a generalized linear model, transforms linear regression values between (0,1) by leveraging a sigmoid equation. This process turns continuous variable regression into a dichotomous task by setting a threshold.
3. Decision Tree utilize a tree structure, offering a simple and efficient classification method commonly used across various applications.
4. KNN, one of the simplest machine learning methods, classifies samples by calculating the distance between the sample and the training data points in the feature space. The sample’s class is determined by the majority class among the K “nearest neighbor” points. KNN is highly accurate and requires no assumptions about data distribution; however, its computational complexity increases with larger datasets due to its reliance on distance calculations.
5. Random Forest is a typical ensemble learning method based on Bagging, suitable for addressing classification and regression problems. By constructing multiple Decision Trees, the model enhances information gain, reducing interference caused by noisy data. The random forest output is determined by the majority of Decision Tree outputs.
6. Gradient Boosting model combines multiple weak models to create more powerful and accurate ones. Gradient Boosting improves prediction accuracy by iteratively enhancing the estimation of weak models.

By utilizing these six machine learning methods, this study aims to identify the most effective method for predicting factors influencing unmet ADL needs among the oldest old with disabilities in China. The results can contribute to the development of public policies and the improvement of caregiver knowledge and skills.

### 2.5. SHAP: increasing interpretability

Machine learning shows impressive predictive capabilities; however, its prediction process often operates as a black box, leading to limited interpretability of the outcomes (39, 40). In other words, numerous complex machine learning algorithms create “black box models” characterized by constrained interpretability. While these models can determine the effectiveness of a specific indicator system, they cannot provide detailed explanations for individual indicators (41). To address this issue, Lundberg and Lee introduced SHAP, a tool designed to enhance the interpretability of machine learning models by calculating the contribution value of each feature and considering it as a contributor to the model’s prediction. The model’s final prediction is obtained by summing the contribution values of all features (42).

Owing to its robust interpretability and visual representation, the SHAP framework has been widely adopted within the machine learning domain. This explanatory model, which aligns with human intuition, has gained increasing popularity in recent years for explaining machine learning models associated with medical diagnoses, social behaviors, and other complex phenomena (43).

### 2.6. Data analysis

Table 1 presents the comparison between oldest old with unmet ADL needs and those without. Continuous variables are compared using a *t*-test, with results presented as mean ± standard deviation. Categorical variables are compared *via* a chi-square test, and the results are reported as the number and percentage. Statistical significance was determined using a two-tailed *p*-value of less than 0.05, indicating that the probability of obtaining the observed results by chance alone is less than 5%.

**Table 1:** Descriptive statistics.

Variables	Oldest old with unmet ADL needs	Oldest old without unmet ADL needs	*p* value
Demographic variables			
Gender			
Female	821 (69.64%)	842 (66.98%)	0.160
Male	358 (30.36%)	415 (33.02%)	
Age	95.63 ± 6.63	96.29 ± 6.68	0.217
Marital status			
No	1,046 (88.72%)	1,115 (88.70%)	0.990
Yes	133 (11.28%)	142 (11.30%)	
Socioeconomic variables			
Household registration			
Rural	859 (72.86%)	713 (56.72%)	<0.001
Urban	320 (27.14%)	544 (43.28%)	
Educational attainment			
Primary and below	836 (70.91%)	768 (61.10%)	<0.001
Middle and above	343 (29.09%)	489 (38.90%)	
Pension			
No	930 (78.88%)	849 (67.54%)	<0.001
Yes	249 (21.12%)	408 (32.46%)	
Economic satisfaction			
No	243 (20.61%)	118 (9.39%)	<0.001
Yes	936 (79.39%)	1,139 (90.61%)	
Economic rank			
Very poor	36 (3.05%)	13 (1.03%)	<0.001
Poor	190 (16.12%)	72 (5.73%)	
Average	830 (70.40%)	824 (65.55%)	
Rich	107 (9.08%)	288 (22.91%)	
Very rich	16 (1.36%)	60 (4.77%)	
Health status variables			
Self rated health			
Very unhealthy	49 (4.16%)	12 (0.95%)	<0.001
Unhealthy	272 (23.07%)	180 (14.32%)	
Average	589 (49.96%)	558 (44.39%)	
Healthy	217 (18.41%)	385 (30.63%)	
Very good	52 (4.41%)	122 (9.71%)	
Disability class			
Low-level	451 (38.25%)	709 (56.40%)	<0.001
Medium-level	439 (37.23%)	396 (31.50%)	
High-level	289 (24.51%)	152 (12.09%)	
Family relationship variables			
Number of children	4.36 ± 1.98	4.47 ± 1.96	0.157
Financial support given to children			
No	312 (26.46%)	334 (26.57%)	0.952
Yes	867 (73.54%)	923 (73.43%)	
Financial support received from children			
No	790 (67.01%)	745 (59.27%)	<0.001
Yes	389 (32.99%)	512 (40.73%)	
Living arrangement			
Otherwise	210 (17.81%)	176 (14.00%)	0.010
Live with children	969 (82.19%)	1,081 (86.00%)	
Caregiving relationship variables			
Perceived control			
Very low	49 (4.16%)	52 (4.14%)	<0.001
Low	177 (15.01%)	146 (11.61%)	
Average	505 (42.83%)	373 (29.67%)	
High	309 (26.21%)	384 (30.55%)	
Very high	139 (11.79%)	302 (24.03%)	
Primary caregiver			
Spouse	83 (7.04%)	75 (5.97%)	0.541
Others	224 (19.00%)	247 (19.65%)	
Children	872 (73.96%)	935 (74.38%)	
Caregiver willingness			
No	198 (16.79%)	46 (3.66%)	<0.001
Yes	981 (83.21%)	1,211 (96.34%)	

In training the machine learning method, samples were first randomly divided into a training set (70%) and a test set (30%). The optimal parameters for each model were then determined using ten-fold cross-validation in the training set. To optimize the machine learning methods, we used a combination of manual parameter tuning, grid search, and random search techniques. Manual parameter tuning involved adjusting model parameters by hand to achieve the best performance, while grid search and random search involved systematically testing different combinations of parameters to find the best combination. Ten-fold cross-validation was chosen as the method for parameter tuning because it provides a good balance between bias and variance in the estimated performance of the model. Performance of the models was assessed using evaluation metrics such as accuracy, precision, recall, F1 score, and AUROC. These metrics provided important information about the accuracy and reliability of the model’s predictions. Because our outcome variable is binary (i.e., unmet ADL needs present or absent), the AUROC is a suitable evaluation metric as it measures the model’s ability to correctly distinguish between positive and negative cases. The calculation formula and explanation for the above model evaluation metrics are as follows.

1. Accuracy. Accuracy measures the proportion of correctly predicted instances (both true positives and true negatives) out of the total instances.

Accuracy = (Number of Correct Predictions) / (Total Number of Predictions).

2. Precision. Precision represents the ratio of true positive predictions to the total predicted positive instances. It quantifies the model’s ability to avoid false positives.

Precision = (True Positives) / (True Positives + False Positives).

3. Recall. Recall calculates the ratio of true positive predictions to the total actual positive instances. It measures the model’s ability to identify all positive instances.

Recall = (True Positives) / (True Positives + False Negatives).

4. F1 score. The F1 score is the harmonic mean of precision and recall. It provides a balanced measure of a model’s performance, especially when dealing with imbalanced datasets.

F1 score = 2 * (Precision * Recall) / (Precision + Recall).

5. AUROC. ROC curves plot the true positive rate (recall) against the false positive rate as the classification threshold changes. AUROC represents the area under this ROC curve and is a good metric for assessing a model’s performance across different threshold values. Higher AUROC values indicate better model performance.

Based on these evaluation indicators, we pinpointed the optimal model for predicting unmet ADL needs among the oldest old with disabilities. The optimal model provides the best balance between performance and interpretability. To understand the importance and influence of each variable on unmet ADL needs, we used SHAP to decompose the contribution of each feature to the prediction results based on the optimal model. SHAP is a useful tool for interpreting machine learning methods because it provides insights into the importance and influence of each variable on the outcome, which can help guide decision-making and policy development. The aforementioned data analysis was conducted using Stata (Stata 17 MP) and Python (Python 3.10) software platforms.^3^

## 3. Results

### 3.1. Descriptive statistics

Table 1 presents the distribution of the 17 predictor variables between the unmet ADL needs present or absent oldest old. Of the total sample of 2,436 oldest old with disabilities, 1,179 (48.4%) reported having unmet ADL needs. Using a chi-square test or t-test as appropriate, significant differences were found in most predictor variables between the two groups, including household registration, educational attainment, pension, economic satisfaction, economic rank, living arrangement, self-rated health, disability class, financial support given to children, perceived control, and caregiver willingness. While there were no significant differences observed for predictor variables such as gender, age, marital status, number of children, financial support received from children, and primary caregiver, these variables may still have relevance for understanding other aspects of care needs and preferences among this population and warrant further investigation in future research.

### 3.2. Performance of machine learning methods

**Figure 2:**
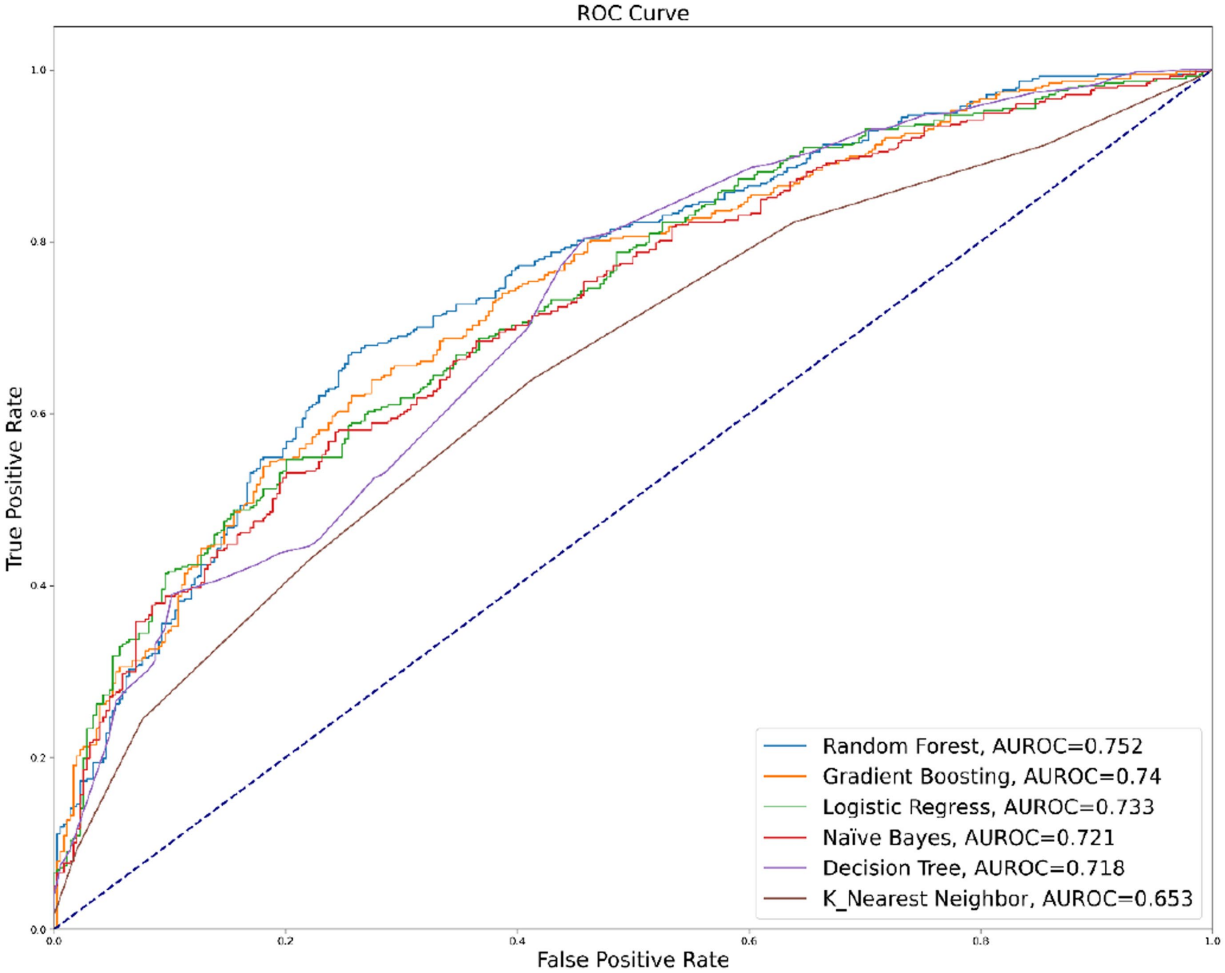
AUROC of Six Machine Learning Models.

Table 2 presents the performance of six machine learning methods in predicting unmet ADL needs among the oldest old with disabilities. All models achieved AUROC values greater than 0.65 (as shown in Figure 2), indicating good predictive performance for unmet ADL needs among the oldest old with disabilities. These results compare favorably to previous studies on the topic and suggest that machine learning algorithms can be effective tools for predicting unmet ADL needs in this population. Among the six models tested, the Random Forest model achieved the highest AUROC (0.752) and good accuracy (0.702), recall (0.660), and precision (0.735) scores, indicating that it was the most effective model for predicting unmet ADL needs among the oldest old with disabilities. The results demonstrate the potential of machine learning methods, particularly the Random Forest model, in predicting unmet ADL needs among the oldest old with disabilities.

**Table 2:** Predictive performance of six machine learning models.

Model	Accuracy	Recall	Precision	F1 score	AUROC
Random Forest	0.702	0.660	0.735	0.696	0.752
Gradient Boosting	0.670	0.658	0.689	0.673	0.740
Logistic Regression	0.651	0.716	0.646	0.679	0.733
Naïve Bayes	0.644	0.782	0.624	0.694	0.721
Decision Tree	0.647	0.700	0.645	0.672	0.718
KNN	0.614	0.639	0.623	0.631	0.653

Table 3 provides the specific parameters for the six models used in this study. In this study, only a small number of parameters were tuned, and the default settings were kept for the rest of the parameters. For instance, the Random Forest tuning parameters used a grid search to determine the optimal parameters n_estimators (n_estimators = 94), max_depth (max_depth = 6), max_features (max_features = 0.1), and min_samples_split (min_samples_split = 9).

**Table 3:** Parameters of six machine learning model.

Model	Parameters
Random forest	n_estimators = 94, max_depth = 6, max_features = 0.1, min_samples_split = 9
Gradient boosting	n_estimators = 97, min_samples_split = 18, min_samples_leaf = 11, max_features = 0.2, max_depth = 7, learning_rate = 0.05
Logistic regression	solver = newton-cg, C = 0.001
Naïve bayes	var_smoothing = 0.1
Decision tree	max_depth = 7, max_features = 15, min_samples_split = 16, min_samples_leaf = 14
KNN	n_neighbors = 9

### 3.3. Feature importance

**Figure 3:**
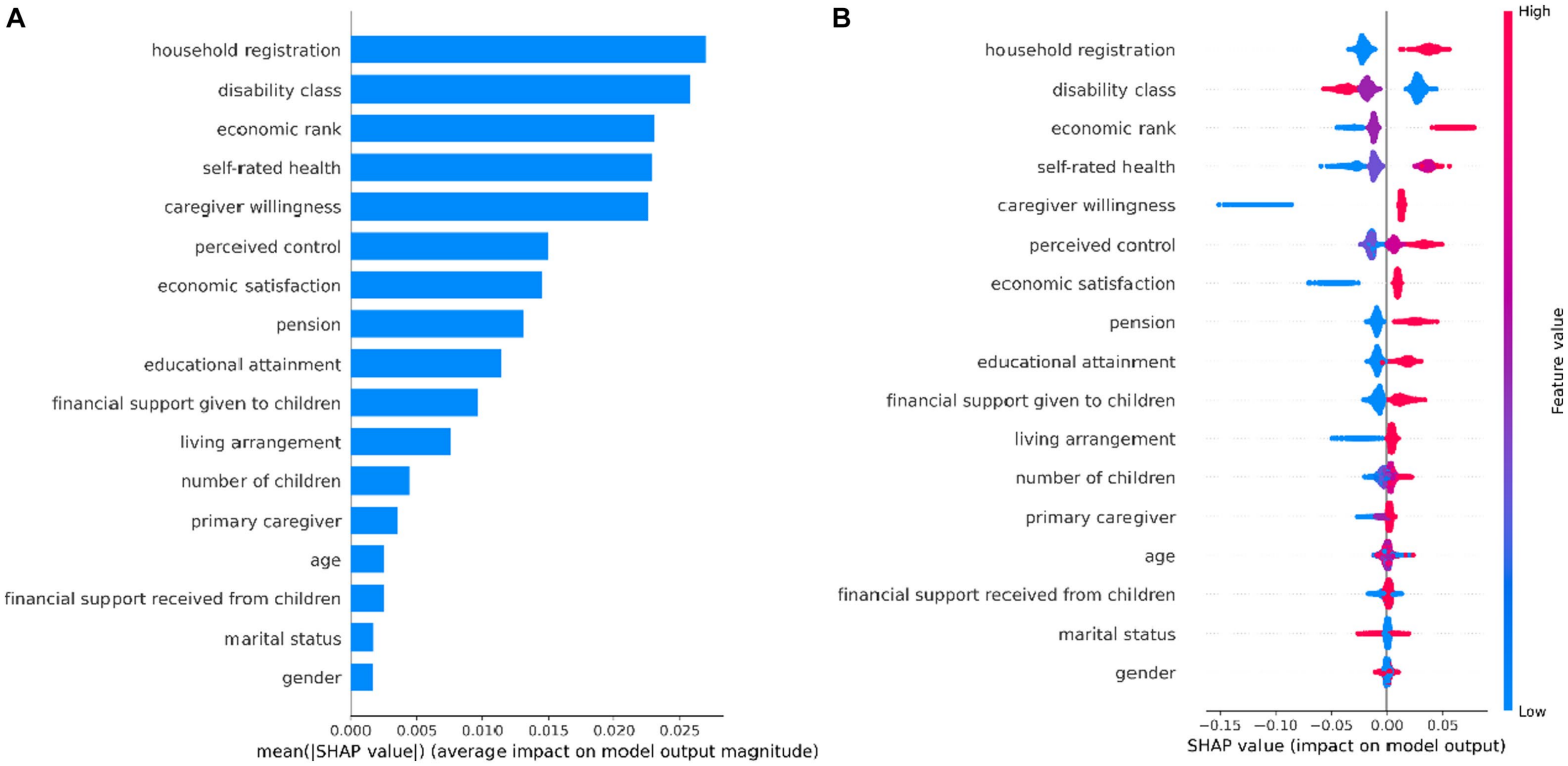
Variable Importance in Random Forest Model. **(A)** Mean SAHP value **(B)** SHAP value.

To gain insights into the factors influencing unmet ADL needs among the oldest old with disabilities, we used SHAP to learn how individual variables contributed to the Random Forest model’s prediction. Figure 3A shows that the factors influencing unmet ADL needs among the oldest old with disabilities, in order of importance, are: household registration, disability class, economic level, self-rated health, caregiver willingness, perceived control, economic satisfaction, pension, educational attainment, financial support given to children, living arrangement, number of children, primary caregiver, financial support received from children, age, gender, and marriage.

As seen in Figure 3B, the likelihood of having unmet ADL needs was higher among the oldest old with the following characteristics: rural household registration, high disability class, low economic class, poor self-rated health, low caregiver willingness, low perceived control, low economic satisfaction, having no pension, low education level, not giving children possessions, living without children, having less children, and having others as primary caregivers. Receiving financial support from children, age, gender, and marriage were not significantly associated with unmet ADL needs among the oldest old with disabilities. These findings provide valuable insights into the factors that influence unmet ADL needs among the oldest old with disabilities. By identifying key factors associated with unmet ADL needs among the oldest old with disabilities, the results of this study can inform the development of targeted interventions and policies aimed at improving the quality of care for this vulnerable population. For example, policymakers may use this information to prioritize funding for support services or programs that address the specific needs of older persons with disabilities and improve their overall care status.

## 4. Discussion

Aging is indeed a prevalent issue faced by societies worldwide, and the likelihood of disability in older person tends to increase with age (43, 44). Providing adequate care for older persons with disabilities is crucial for maintaining their dignity and quality of life. In this context, identifying the factors affecting unmet ADL needs and developing appropriate responses is essential.

Existing studies have extensively explored this issue using linear models (12–14). However, these models have limitations in identifying the nonlinear effects of variables, and the magnitude of variable coefficients may not accurately measure the relative importance of variables. Additionally, many studies have identified variables that influence outcomes without prioritizing their importance (12, 13). In the context of limited care resources, determining the relative importance of factors affecting unmet needs is a necessary step to optimize resource utilization. Machine learning algorithms, such as the Random Forest model, can help address these limitations by identifying complex relationships between variables and ranking their relative importance. This enables policymakers and care providers to prioritize interventions and allocate resources more effectively, ultimately improving the quality of life for older persons with disabilities.

Machine learning is an important branch of artificial intelligence. In the era of complex big data, machine learning plays an increasingly crucial role in the field of geriatric health due to its powerful predictive capabilities. For example, Xin and Ren analyzed the main factors influencing depression among older persons with disabilities in rural and urban China using the Random Forest model (45). Lin et al. employed multiple machine learning methods to analyze depression factors in a home-based older population (46). Kitcharanant et al. utilized machine learning to analyze mortality in older persons within 1 year after experiencing a fragility hip fracture (47). These studies demonstrate the potential of machine learning to improve prediction and analysis in geriatric health.

Similarly, in this study, the machine learning model achieved impressive results. Six machine learning models - Naive Bayes, Logistic Regression, Decision Tree, KNN, Random Forest, and Gradient Boosting - were employed in the analysis. The AUROC values for all models were greater than 0.65, indicating good prediction performance. Among them, the Random Forest model had the best prediction with an AUROC of 0.752, accuracy of 0.702, recall of 0.660, and precision of 0.735. The strong predictive power of the machine learning models in this study is consistent with the results of previous studies, which have demonstrated the potential of machine learning algorithms in predicting the outcomes of interest in geriatric health research (45–47).

To further investigate the factors influencing unmet ADL needs among the oldest old with disabilities, this study used SHAP based on the Random Forest model with the optimal predictive performance. The results identified household registration, disability class, economic status, self-rated health, caregiver willingness, perceived control, economic satisfaction, pension, educational attainment, financial support given to children, living arrangement, number of children, and primary caregiver as the factors influencing unmet ADL needs among the oldest old with disabilities, in order of importance. These findings provide valuable insights into the factors that contribute to unmet ADL needs in this population and can guide policymakers and care providers in developing targeted interventions and allocating resources effectively. It is noteworthy that receiving financial support from children, age, gender, and marriage were not significantly associated with unmet ADL needs among older persons with disabilities. Our findings are consistent with some studies, but there are also differences.

The association between household registration and unmet ADL needs is consistent with the study by Zhu and Oesterle (14), who found a significant association between urban and rural household registration on unmet needs in China. Disabled people with rural household registration were more likely to have unmet needs than those with urban household registration. Hu and Wang found older people living in rural communities have a higher level of unmet needs than those in urban communities (19). In addition, Chen et al. also found rural older females are more likely to experience unmet needs compared with urban counterparts (36). Possible reasons for this include the large exodus of young laborers from rural China, leading to spatial distancing of offspring from older persons (48), and a dramatic shrinkage of family caregiving capacity. Additionally, weaker affordability and accessibility of formal care for rural residents make them less likely to receive formal care (14, 49).

The study by Schure et al. has also found an association between the disability class and unmet ADL needs (50). Individuals with more severe disabling conditions are more likely to experience unmet ADL needs, and the determinants of inadequate care versus no care differed with respect to disability class (21). Hu and Wang found disability class affects both the likelihood and the level of unmet needs (19). This result is not surprising, given the strong correlation between disability status and the level of ADL needs. Similar to the disability class, self-rated health is a significant predictor variable of unmet ADL needs. This finding is consistent with Zhu’s study (13). In Peng et al.’s study, having good self-rated health also reduced the risk of unmet care (12). The logic behind this association is that the poorer the self-rated health of older persons with disabilities, the stronger the need for ADL assistance, and the higher the likelihood of unmet ADL needs.

In addition to household registration, the socioeconomic dimension factors of economic rank, economic satisfaction, pension, and educational attainment were all significantly associated with unmet ADL needs. Desai et al. also have found an association between economic status and unmet ADL needs (51). Zhu’s study also revealed that the risk of unmet long-term needs largely depends on the economic status of the oldest old (13). But in Momtaz et al.’s study, household income was not found to be a significant predictor of unmet need (22). This difference may be due to differences in country contexts. Given the substantial resources required for long-term care, individuals in poorer economic status are more likely to experience unmet need. At the same time, this also reflects, to some extent, a shift in China’s care system, where money has naturally become a major factor in unmet ADL needs as family caregiving capacity has shrunk.

Apart from objective health and financial status, caregiver willingness and perceived control, which reflect the caregiving patterns of the older persons with disabilities, are important variables in predicting unmet ADL needs. Existing studies also found a significant association between caregiver willingness and unmet needs (12, 13). Caregiver willingness affects the quality of caregiving services, which in turn affects the unmet ADL needs among the older persons with disabilities. Perceived control, which allows the older persons with disabilities to make requests that are more in line with their care needs, was found to be significantly associated with unmet ADL needs in this study. This highlights the importance of taking into account the preferences and needs of older persons with disabilities in caregiving, which may reduce the risk of unmet ADL needs.

In terms of the family relationship, this study found that providing financial support to children was significantly associated with unmet caregiving needs among the oldest old with disabilities. This finding aligns with the intergenerational exchange theory, where providing financial support to children is associated with better caregiving services. In Chen et al.’s study, higher income levels for caregivers also reduced the likelihood of unmet care need (36). Moreover, the number of children was also significantly associated with unmet ADL needs. This finding is consistent with Peng et al.’s study (12). Lima and Allen’s research also shows that availability of social support were key factors related to a situation of no care (21). Older persons with more children tend to have greater access to family caregiving resources and are less likely to have unmet ADL needs.

Interestingly, this study found weak associations between age, marital status, and gender with unmet ADL needs, which is inconsistent with Chen et al.’s findings (36). Momtaz et al. found that age was not a significant predictor of unmet needs (22). In Peng et al.’s study, age and marital status were also not significantly associated with unmet needs (12). These may be attributed to the fact that the sample population in this study consisted of older persons with disabilities aged 80 years and above, where the biological factors of age and gender might have less influence on their health. Additionally, it can be challenging for older persons’ spouses to provide effective caregiving support.

Compared to previous studies, this study offers the following advantages: Firstly, it introduces machine learning to analyze factors affecting unmet ADL needs among the oldest old with disabilities. Machine learning models offer stronger predictive power without requiring additional model assumptions, unlike traditional linear models. Secondly, the study combines six machine learning algorithms, including Naïve Bayes, Logistic Regression, Decision Tree, KNN, Random Forest, and Gradient Boosting, to ensure robust results. Among these models, Random Forest demonstrated the most effective predictive performance. Lastly, by combining machine learning with SHAP, this study builds an interpretable model, allowing for a thorough exploration of the importance and influence of different factors on unmet ADL needs among the oldest old with disabilities.

However, this study has some limitations that should be acknowledged. Firstly, the context of the study is limited to China, and the findings may not be generalizable to other countries due to the unique cultural, social, and economic characteristics of China. For instance, while household registration is a significant predictor of unmet ADL needs among the oldest old with disabilities in China, it may have limited relevance for other countries due to its unique nature. Secondly, this study focuses on the oldest old with disabilities, which may not reflect the situation of younger older persons with disabilities. While age and marital status were not predictor variables affecting unmet ADL needs among the oldest old with disabilities, this may differ for the younger old group. Lastly, the study did not include all possible factors that may influence unmet ADL needs, and future research should explore additional variables to gain a more comprehensive understanding of the factors affecting unmet ADL needs among the oldest old with disabilities. Despite these limitations, this study provides valuable insights into the factors associated with unmet ADL needs among the oldest old with disabilities, which can inform policies and interventions aimed at improving the quality of life for this vulnerable population.

Our study on predicting unmet ADL needs among the oldest old with disabilities in China holds crucial policy implications. Firstly, optimizing resource allocation is paramount to address disparities in access to care and support. The factors of household registration and economic rank were found to be associated with unmet ADL needs. Policymakers must focus on equitable distribution of resources, ensuring that economically disadvantaged and remote regions have adequate support and services available to meet the needs of older persons with disabilities.

Secondly, tailored care and support for individuals with different disability classes are essential to address their specific requirements. Customized rehabilitation programs and welfare measures must be implemented to cater to the diverse needs of each disability group. By acknowledging and addressing these unique needs, policymakers can enhance the overall well-being and quality of life for the oldest old with disabilities in China.

Thirdly, to bolster the overall support system, policymakers should emphasize the importance of economic security for older adults. Improving pension schemes and providing additional financial aid to those in need can significantly reduce financial burdens and enable older individuals to better cope with their ADL requirements. This approach will foster a more dignified and supportive environment for the oldest old with disabilities.

In conclusion, addressing unmet ADL needs among the oldest old with disabilities in China requires a multifaceted approach. Policymakers must optimize resource allocation, provide tailored care and support, and strengthen economic security to enhance the well-being and quality of life of this vulnerable population. By implementing these policy measures, the government can create a more inclusive and supportive society for the oldest old with disabilities, fostering their independence and overall happiness in their later years.

## 5. Conclusion

This study employed six machine learning methods to predict unmet ADL needs among the oldest old with disabilities in China, with the Random Forest algorithm demonstrating the highest prediction accuracy. The analysis identified critical factors influencing unmet ADL needs, emphasizing the importance of addressing socioeconomic disparities, health status, and caregiving relationship factors. To alleviate unmet ADL needs, our findings suggest that government interventions should focus on bridging the urban–rural divide, supporting those with deteriorating health status, and enhancing caregiver skills and resources. The study’s results can guide policymakers and stakeholders in designing and implementing effective interventions to improve the well-being and quality of life for the oldest old with disabilities in China. Furthermore, the machine learning approach showcased in this study can be adapted and utilized in other countries and regions to better understand and address the unmet ADL needs of their ageing populations.

## Data availability statement

The original contributions presented in the study are included in the article/supplementary material, further inquiries can be directed to the corresponding author.

## Ethics statement

The ethics committee of the Institutional Review Board, Duke University (Pro00062871), and the Biomedical Ethics Committee, Peking University (IRB00001052–13074) approved all the procedures. All methods were performed in accordance with the relevant guidelines and regulations. Informed consent was obtained from both the literate participants and the legal guardian/next of kin of illiterate participants.

## Author contributions

KW: Writing – original draft, Writing – review & editing, Conceptualization, Data curation, Formal Analysis, Investigation, Methodology, Software, Validation, Visualization. JZ: Writing – original draft, Writing – review & editing, Visualization. JH: Writing – original draft, Writing – review & editing, Methodology. DL: Writing – review & editing, Visualization. YL: Writing – review & editing, Conceptualization, Formal Analysis, Funding acquisition, Project administration, Supervision.

## Funding

The author(s) declare that no financial support was received for the research, authorship, and/or publication of this article.

## Conflict of interest

The authors declare that the research was conducted in the absence of any commercial or financial relationships that could be construed as a potential conflict of interest.

## Publisher’s note

All claims expressed in this article are solely those of the authors and do not necessarily represent those of their affiliated organizations, or those of the publisher, the editors and the reviewers. Any product that may be evaluated in this article, or claim that may be made by its manufacturer, is not guaranteed or endorsed by the publisher.

## Footnotes

^1^Chart: Key Data from the Seventh National Population Census. The Central People’s Government of the People’s Republic of China. http://www.gov.cn/xinwen/2021-05/11/content_5605871.htm

^2^We also defined the oldest old as individuals aged 85 and above for the study, and the results did not show significant changes.

^3^GitHub link to the machine learning script used in this study: https://github.com/wangkun543604/predictADLneeds.git.

## Abbreviations

ADL: activities of daily living
CLHLS: Chinese Longitudinal Healthy Longevity Survey
SHAP: Shapley Additive exPlanations
KNN: K-Nearest Neighbor
AUROC: Area Under the Receiver Operating Characteristic Curve
